# The NEON Daily Isotopic Composition of Environmental Exchanges Dataset

**DOI:** 10.1038/s41597-022-01412-4

**Published:** 2022-06-21

**Authors:** Catherine E. Finkenbiner, Bonan Li, Lindsey Spencer, Zachariah Butler, Marja Haagsma, Richard P. Fiorella, Scott T. Allen, William Anderegg, Christopher J. Still, David Noone, Gabriel J. Bowen, Stephen P. Good

**Affiliations:** 1 Department of Biological and Ecological Engineering, Oregon State University, Corvallis, USA; 2 Water Resources Graduate Program, Oregon State University, Corvallis, USA; 3 Department of Geology and Geophysics, University of Utah, Utah, USA; 4 Earth and Environmental Sciences Division, Los Alamos National Laboratory, New Mexico, USA; 5 Department of Natural Resources and Environmental Science, University of Nevada-Reno, Reno, USA; 6 Department of Biology, University of Utah, Utah, USA; 7 Department of Forest Ecosystems and Society, Oregon State University, Corvallis, USA; 8 Department of Physics, University of Auckland, Auckland, New Zealand

**Keywords:** Hydrology, Ecology

## Abstract

The National Ecological Observatory Network (NEON) provides open-access measurements of stable isotope ratios in atmospheric water vapor (δ^2^H, δ^18^O) and carbon dioxide (δ^13^C) at different tower heights, as well as aggregated biweekly precipitation samples (δ^2^H, δ^18^O) across the United States. These measurements were used to create the NEON Daily Isotopic Composition of Environmental Exchanges (NEON-DICEE) dataset estimating precipitation (P; δ^2^H, δ^18^O), evapotranspiration (ET; δ^2^H, δ^18^O), and net ecosystem exchange (NEE; δ^13^C) isotope ratios. Statistically downscaled precipitation datasets were generated to be consistent with the estimated covariance between isotope ratios and precipitation amounts at daily time scales. Isotope ratios in ET and NEE fluxes were estimated using a mixing-model approach with calibrated NEON tower measurements. NEON-DICEE is publicly available on HydroShare and can be reproduced or modified to fit user specific applications or include additional NEON data records as they become available. The NEON-DICEE dataset can facilitate understanding of terrestrial ecosystem processes through their incorporation into environmental investigations that require daily δ^2^H, δ^18^O, and δ^13^C flux data.

## Abstract (summary)

**Table:** 

Measurement(s)	stable isotope ratios in carbon dioxide • stable isotope ratios in atmospheric water vapor • stable isotope ratios in precipitation
Technology Type(s)	eddy covariance towers • wet deposition collector
Factor Type(s)	location
Sample Characteristic - Environment	climate system
Sample Characteristic - Location	United States of America

## Background & Summary

The stable isotope ratios of carbon and water fluxes are natural environmental tracers that can be used to provide new insights into hydrological, ecological, and meteorological processes, as well as provide supportive metrics for understanding the complex feedbacks between the land surface and atmosphere^1–6^. These tracers are informative in ecohydrologic modeling applications because they provide additional points of comparison between observed and modeled environmental pools and fluxes^7^, and provide a benchmark with which to evaluate the performance and efficiency of modeling approaches^8^.

Tracers commonly used in studies of land surface processes from global to local scales include the stable isotope ratios of hydrogen (^2^H/^1^H), oxygen (^18^O/^16^O), and carbon (^13^C/^12^C), found in water and carbon dioxide, hereafter expressed as δ^2^H, δ^18^O and δ^13^C values^8–13^. Water isotope ratios provide useful information for partitioning evapotranspiration (ET) into evaporation and transpiration at the ecosystem scales^3,14^, as well as understanding water use efficiency in forests, agricultural, and other ecosystems^15,16^. Carbon isotope ratios provide valuable information about the component fluxes that determine net ecosystem exchange (NEE) of carbon dioxide between ecosystems and the atmosphere^13,17^. Insights into such processes support the understanding of plant water uptake strategies and the underlying principles of water, carbon, and energy cycling in the soil-vegetation-atmosphere continuum^3,14,18,19^.

The United States National Ecological Observatory Network (NEON) collects long-term ecological data in eco-climatologically diverse field sites across the United States and provisions these data through an open-access data portal (https://data.neonscience.org/). These publicly available isotope datasets are part of an important network documenting hydrometeorological tracer patterns throughout North America. The NEON atmospheric gas stable-isotope measurements are collected at different tower heights, with each height being sampled approximately hourly (varying by site), enabling robust daily calculations of NEE and ET flux isotope ratios (e.g., via mass balance approaches developed by Keeling^20^ and Miller-Tans^21^). The precipitation isotope data are collected at biweekly intervals, but they can be downscaled to a daily resolution using a validated approach^22^. By conducting those pre-processing steps, we can facilitate subsequent applications using these published daily flux data products.

We generated daily records of a) δ^2^H and δ^18^O in precipitation fluxes (*F*_*P*_) at 25 NEON core sites and 19 NEON gradient sites, b) δ^2^H and δ^18^O of ET fluxes (*F*_*ET*_) at 19 NEON core sites and 2 NEON gradient sites, and c) δ^13^C of NEE fluxes (*F*_*NEE*_) at 19 NEON core sites and 28 NEON gradient sites. These products form the NEON Daily Isotopic Composition of Environmental Exchanges (NEON-DICEE) datasets characterizing flux isotope ratios across diverse ecosystems over a multi-year span created using consistent instrumentation and methodology. The NEON-DICEE datasets provided here are reproducible with the published python and R scripts. The methods can be modified to fit a user’s specific application or as additional NEON data records become available in the future. The subsequent sections detail the methods used to derive isotope ratios associated with the *F*_*P*_, *F*_*ET*_ and *F*_*NEE*_ fluxes, the validation of each method, and a description of the associated metadata.

## Methods

### Precipitation flux data products

We acquired 30-minute precipitation amount data and stable water isotope ratios in precipitation collected in biweekly intervals from the NEON Data Portal^23^ using the neonUtilities R package^24^. A wet deposition collector at each site opens during rain events to collect samples. These precipitation event samples were combined to create a 2-week water composite which are then retrieved, filtered, and analyzed^25^. All datasets with stable water isotopes in precipitation (as of February 2022) were downloaded, as well as the corresponding precipitation amount data. There were 44 sites across the NEON network with water isotope data, 38 of which contained sufficient data to perform the downscaling methodology (refer to the NEON data product number DP1.00006.001 for precipitation amounts and DP1.00038.001 for precipitation stable isotope ratios). At the time of this publication, NEON’s “primary” precipitation data had a documented issue where the weighing gauge had recorded spurious small precipitation amounts^25^. Consequently, we used the “secondary” collector data (from a tipping bucket rain gauge) when available, and the primary data was only used at sites that did not have secondary data. As a further quality check, we removed trace precipitation events, defined here as less than 0.25 mm of accumulated precipitation in one day. This threshold can be changed within the code to generate new data products.

The NEON precipitation samples for isotope analysis are collected at a biweekly resolution. This temporal resolution is higher relative to past network collections (e.g., the Global Network of Isotopes in Precipitation (GNIP) contains data mostly at monthly resolutions^26^) but remains coarser than what is ideal for land modelling applications. Consequently, by downscaling from stable isotope analyses on the bi-weekly precipitation composites to daily resolution a fine-scale product is available for future studies. The daily stable water isotopes associated with the *F*_*P*_ were generated at each NEON site with sufficient observation data to conduct a statistical downscaling method^22^, which translated the observed biweekly water isotope samples to a daily estimate correlating with known precipitation amounts. The downscaling method was run 100 times to create an ensemble of *F*_*P*_ timeseries with associated isotope ratios (δ^2^H and δ^18^O) to characterize the range of expected values. We provide all 100 ensemble realizations, as well as ensemble summary statistics (mean and standard deviation). The statistical downscaling method was described in detail and validated by Finkenbiner *et al*.^22^, here we provide a summary of the method applied to the NEON datasets.

At each NEON site, the 30-minute precipitation amounts were aggregated to daily and biweekly totals to correspond with the biweekly tracer observations and provide a daily time series on which to condition the generated daily tracer values. The seasonal component of each δ^2^H and δ^18^O time series was characterized using a combination of sinusoidal functions through Fourier decomposition, following the methods from Allen *et al*.^27^, and removed. The remaining isotope values were assumed to be drawn from a purely stochastic process with a mean of zero. The daily covariance statistics were predicted based on trends in the means, standard deviations, and Pearson correlation coefficients as each time series was aggregated from biweekly to coarser resolutions. Some NEON sites had few biweekly observations and were unable to be aggregated to the 12-week resolution required for the downscaling method. We anticipate as NEON continues to collect observations and the site data represents longer seasonal time scales, this downscaling method can be applied at additional sites. δ^2^H and δ^18^O stable water isotopes are strongly correlated with each other^28^ and often share a weaker, but significant, relationship with precipitation amount^29^. A Gaussian copula^30^ conditioned on daily precipitation amounts was used to generate pseudo-random values from the predicted de-seasonalized daily statistics of δ^2^H and δ^18^O from the stochastic signal. A copula is a multivariate cumulative distribution function used to model the dependence between random variables^30^, here the random variables were precipitation amount and its isotopic ratios. Lastly, the pre-defined seasonal component was added to each stochastic isotope series and a residual correction was performed. The residual correction adjusts the downscaled daily δ^2^H and δ^18^O values by forcing the biweekly precipitation weighted means of the downscaled data to match those of the observed biweekly dataset. At each NEON site with sufficient observation data, we generated 100 final synthetic daily time series which corresponded with daily precipitation amounts, seasonal signals, and stochastic variability.

### Gas flux data products

The NEON atmospheric isotope measurements provide continental-scale ongoing measurements of δ^13^C-CO_2_, δ^18^O-H_2_O_vapor_, and δ^2^H-H_2_O_vapor_ at established eddy covariance towers. The NEON eddy covariance bundled product^31^ (refer to NEON data product number DP4.00200.001) provides δ^2^H, δ^18^O, and δ^13^C values of the atmospheric gases at different tower heights with a typical averaging interval of 9-minutes (the return interval varies and can be up to 90-minutes). Gases are sampled at each height for ten minutes, with the first minute being discarded, and then cycled to the next height; thus, the sampling interval depends on the number of heights sampled per tower. These measurements were used to estimate the stable water and carbon isotope ratios associated with the daily *F*_*ET*_ and *F*_*NEE*_ fluxes.

The estimation of the daily carbon and water vapor isotope composition of the *F*_*ET*_ and *F*_*NEE*_ fluxes requires calibration of NEON’s measured carbon and water isotope ratios to known field standards that are themselves calibrated to the Vienna Pee Dee Belemnite (VPDB) and Vienna Standard Mean Ocean (VSMOW) scales. The measured carbon and water isotope ratios frequently diverge from the VPDB and VSMOW scales due to the instrumentation drift, poor instrument calibration, or bias that has not yet been removed from the calibration routines. Fiorella *et al*.^13^ provided a calibration strategy to correct the measured δ^13^C values to the VPDB scale as part of the NEONiso R package^24^. Since that publication, NEONiso has been expanded to include calibrations for δ^2^H and δ^18^O values. We estimated the isotopic composition of the surface fluxes relative to the calibrated atmosphere fluxes by employing the Miller-Tans^21^ mixing model approach, which regresses the product of H_2_O or CO_2_ mixing ratios and the isotope composition of each atmospheric gases at each of the measurement heights against the respective mixing ratio for that gas^32^. The slope of the simple regression from this procedure estimated the isotope composition of the water and carbon fluxes. To estimate the daily isotopic value of each flux, we used the 9-minute calibrated isotope datasets across all measurement heights as the predictor of a Miller-Tans mixing model and the response was the product of the 9-minute atmospheric gas concentrations and the calibrated isotope data. We used isotope ratios of the atmospheric gases to create flux data for three time-windows per day: all day, daytime, and nighttime. For daytime data, we only used isotope ratios which occurred during time periods where the incoming shortwave radiation was ≥10 Wm^−2^. In contrast, for nighttime data we only used isotope ratios during periods where the incoming shortwave radiation was <10 Wm^−2^. The 10 Wm^−2^ is based on the standard threshold used in common eddy covariance pre-processing software^33^ where it is assumed periods with ≥10 Wm^−2^ of shortwave radiation only occurred during the daytime. This value can be adjusted using the provided scripts.

While we achieved reasonable results with the Miller-Tans approach, other mass balance mixing methods can be explored in future work to estimate the isotope composition of the water and carbon fluxes (e.g., Zobitz *et al*.^34^, Wehr and Saleska^35^). Laser-based water vapor isotope analysers have known low-vapor measurement deviations^36,37^ below a dry mole fraction of ~5000 ppm (and major below ~2000ppm). At the time of this publication, the water vapor isotope measurements provided by NEON were not corrected for low humidity deviations and the data required to correct for low humidity was not available across the network. Fortunately, most affected periods were during the winter at cold sites, where ET is expected to be low, but future work could apply these corrections to the data.

## Data Records

NEON-DICEE associated data, code and metadata are available in a public repository on HydroShare^38^. HydroShare, developed and maintained by the Consortium of Universities for the Advancement of Hydrologic Science, Inc. (CUAHSI) and supported by the U.S. National Science Foundation, is a web-based hydrologic information system that allows users to publish and share data repositories in a citable manner.

### Precipitation flux data products

For the daily *F*_*P*_, there are four data files containing time series of modeled δ^2^H and δ^18^O values corresponding to observed precipitation. Two are the mean of modeled δ^2^H and δ^18^O values from the ensemble output and the other two data files are the standard deviations of the modeled δ^2^H and δ^18^O. There is also a metadata file describing the quality of each data point. Each row in these files represents one calendar date and the columns are labeled with the NEON site abbreviation codes. Each column contains a daily time series of water isotope values where applicable. Days with no precipitation contain values of −9999. The precipitation flux metadata file contains quality flags for each value in a site’s time series. A “0” indicates daily isotope value was associated with observed precipitation that was larger than the defined value for trace events (here 0.25 mm), a “1” indicates there was no precipitation data on that day, and a “2” indicates the observed precipitation was less than the defined value for trace events.

**Fig. 1:**
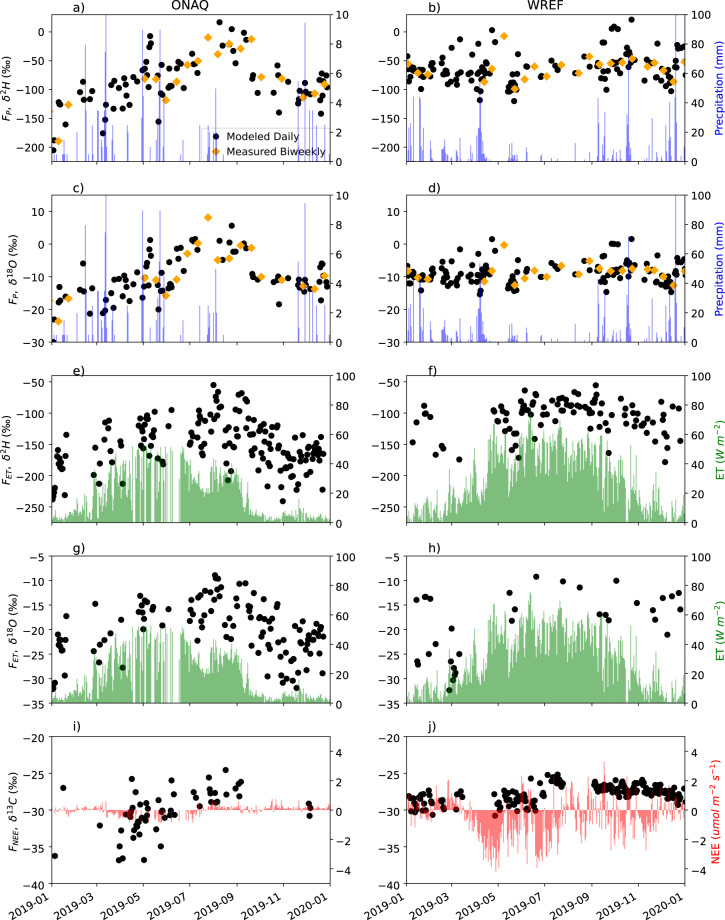
Example NEON-DICEE time series at NEON sites Onaqui, UT (ONAQ - arid climate) and Wind River, WA (WREF - wet climate) of the (**a**–**d**) downscaled daily and observed biweekly ratio of the flux in precipitation (*F*_*P*_) and precipitation amount and (**e**–**j**) daily isotope ratios of the ET and NEE water and carbon fluxes, along with Evapotranspiration (*F*_*ET*_), and Net Ecosystem Exchange (*F*_*NEE*_) fluxes for January 1, 2019 - January 1, 2020.

**Fig. 2:**
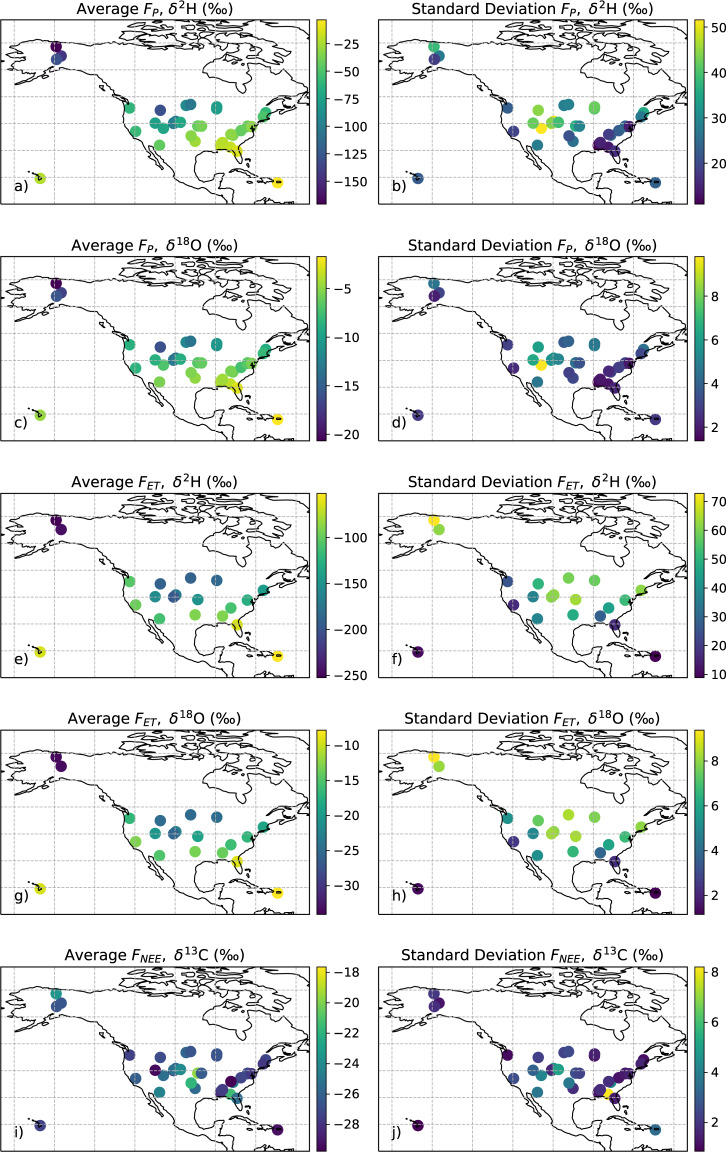
Average and standard deviation of each of NEON-DICEE time series from each of the NEON site locations with (**a**–**d**) precipitation flux (*F*_*P*_) water isotopes (one time series), (**d**–**h**) flux tower ET (*F*_*ET*_) estimates of water isotopes (“all time”), and (**i**,**j**) NEE (*F*_*NEE*_) of carbon isotopes (“all time”).

The precipitation downscaling techniques were successfully applied to sites with low (e.g., Onaqui in Utah (ONAQ)) and high (e.g., Wind River in Washington (WREF)) annual precipitation (Fig. 1a–d). Across all sites which could be statistically downscaled, an average of 23 biweekly observations were used in the downscaling method. The means and standard deviations of the downscaled precipitation isotope values varied depending on site-specific characteristics (e.g., seasonality, climate) and this was accounted for within the downscaling methodology (Fig. 2a–d). The metadata document recorded all sites with sufficient isotope observations. The site with the minimum and maximum number of simulated daily isotope ratios was 146 days (5% of total days) and 1679 days (54% total days), respectively.

### Gas flux data products

Per time-window (all time, daytime, nighttime), water isotope ratios (δ^2^H, δ^18^O) of the *F*_*ET*_ and carbon isotope ratios (δ^13^C) of the *F*_*NEE*_ are contained in three data files with row names consisting of the calendar date and column names corresponding to the four-letter abbreviation of each NEON site location (same as the *F*_*P*_ file format described above). The associated ‘error’ files contain the uncertainty of the estimated flux. This uncertainty is the standard error of the slope coefficient in the simple linear regression of the Miller-Tans mixing model. The included *F*_*ET*_ and *F*_*NEE*_ metadata files contain data quality flags for each flux measurement. For each stable isotope time series, a value of −9999 was used to fill days when no calibrated isotope ratios were available, and this was marked with a “1” quality flag in the metadata file. A flag value of “2” was used if there was a low number (*n* ≤ 5) of calibrated isotope values used to generate the daily flux values. A quality flag value of “3” was assigned when the Miller-Tans mixing model r-squared (R^2^) was below 0.9. To identify cases where the interred point falls outside the range of observations, an interquartile (IQR) range flag was applied to each time series meeting the above criteria (*n* ≥ 5, R^2^ ≥ 0.9); if the inferred isotope value of the flux was beyond the 1.5 times IQR of the 25th percentile (Q_1_-1.5 IQR) and 75th percentile (Q_3_ + 1.5 IQR) of the observation data, then a flag of “4” was assigned to those data points. A “0” quality flag was used to indicate a good isotope value of the flux estimated from the isotope ratios of the gases where *n* ≥ 5 from the regression analysis, R^2^ ≥ 0.9, and the isotope value was within the desired interquartile. Users of these data products are encouraged to use the isotope values in the time series with a quality flag value of “0”. The isotope ratios of the ET and NEE fluxes which passed all quality flags at ONAQ and WREF were shown in Fig. 1e–j, along with the fluxes themselves.

The site averaged (±standard deviation) all time isotope flux values ranged from −252.4 (72.8) ‰ to −51.9 (8.9) for δ^2^H_ET_, −34.1 (9.3) ‰ to −7.8 (1.2) ‰ for δ^18^O_ET_, and −29.8 (1.9) ‰ to −17.6 (1.1) ‰ for δ^13^C_NEE_ (Fig. 2e–j). The site averaged (±standard deviation) daytime isotope flux values ranged from −236.6 (61.9) ‰ to −51.4 (9.5) for δ^2^H_ET_, −32.6 (10.7) ‰ to −7.7 (1.3) ‰ for δ^18^O_ET_, and −32.8 (7.3) ‰ to −18.8 (1.2) ‰ for δ^13^C_NEE_. The site averaged (±standard deviation) nighttime isotope flux values ranged from −262.4 (75.7) ‰ to −50.5 (9.5) for δ^2^H_ET_, −34.3 (9.8) ‰ to −7.5 (1.2) ‰ for δ^18^O_ET_, and −30.1 (1.8) ‰ to −17.2 (1.3) ‰ for δ^13^C_NEE_. Across all NEON sites, the number of δ^18^O_ET_ and δ^2^H_ET_ flux values that passed all quality flags ranged from 127 to 829 days (7 to 45% of total days) and 197 to 1046 days (11 to 56% of total days), respectively. The number of δ^13^C_NEE_ data points that passed all quality flags ranged from 43 to 1324 days (2% to 71% of total days). However, calculated flux isotope ratios that did not pass quality flags might not necessarily be low quality. For example, atmospheric conditions could be such that a two-member mixing model does not work. In this case, the raw data would be of high quality, but the Miller-Tans mixing model method would fail. Future work could investigate other methods besides the Miller-Tans method as to increase the number of data points that pass imposed quality flags.

## Technical Validation

### Precipitation flux data products

**Fig. 3:**
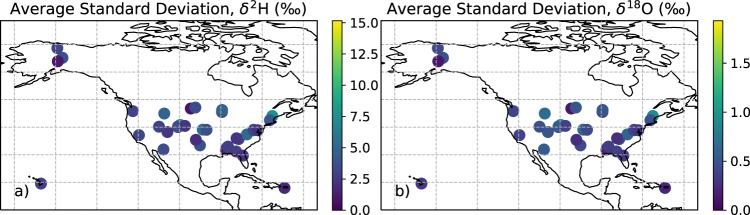
Standard deviation of NEON-DICEE precipitation flux’s (*F*_*P*_) isotope ratio averaged across 100 daily time series generated at each of the NEON site locations. Each point represents the average standard deviation for each day in the time series across 100 downscaled realizations. The maximum expected standard deviation on any given day ranged from 0 to 15.2‰ for δ^2^H and 0 to 1.9‰ for δ^18^O.

The implemented statistical downscaling method was previously validated^22^ at 27 globally distributed sites from the International Atomic Energy (IAEA) Global Network of Isotopes in Precipitation (GNIP) database^26^. Downscaling biweekly observations to daily estimates cannot produce the true value (as these are unknown) but only statistically representative realizations. We quantified the uncertainty associated with the average of the daily precipitation estimates by calculating the standard deviation of each precipitation event across an ensemble of 100 precipitation time series at each site (Fig. 3). The average standard deviations ranged from 2.4 to 9.5‰ for δ^2^H values and 0.3 to 1.6‰ for δ^18^O values across sites. Sites with larger seasonal variability have larger standard deviations. The dataset provided here contains 100 realizations of the estimated daily precipitation’s isotopic composition based on the statistics of a coarser resolution observation time series (here biweekly observations). Depending on the application of the daily isotope ratios, generating an ensemble of daily time series may be advantageous to capture impacts of the standard error as a function of time or space. To generate ensemble sets, the provided python script, which generates a single random realization of the daily precipitation water isotope data product, can be run numerous times.

### Gas flux data products

**Fig. 4:**
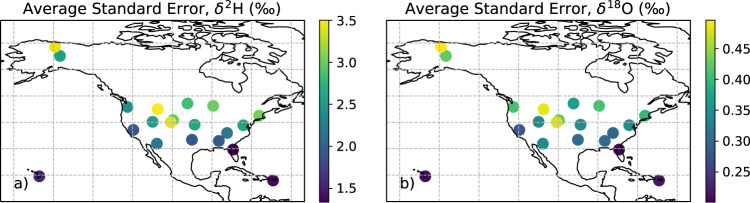
Standard error of the flux tower NEON-DICEE ET (*F*_*ET*_) estimates of water isotopes averaged across time at each NEON site.

**Fig. 5:**
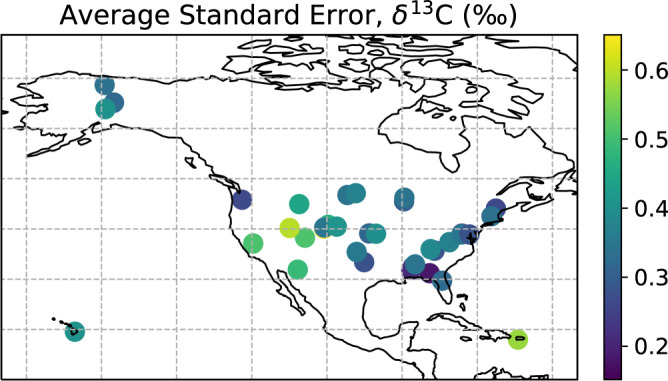
Standard error of the flux tower NEON-DICEE NEE (*F*_*NEE*_) estimates of carbon isotopes across time at each NEON site.

The generation of calibrated isotope ratios associated with the atmospheric carbon dioxide flux were validated by Fiorella *et al*.^13^ (with a bias of 0.11‰ and precision of ~0.4‰) and here similar procedures were used for the atmospheric water vapor ratios. Within the context of a simplified mixing model source estimation approach, uncertainty in the estimation of the isotope composition of surface-atmosphere fluxes is associated with accuracy of isotope measurements themselves and the range of atmospheric carbon dioxide and water vapor concentrations observed during the averaging interval^32^. The average of the daily standard error in the mixing model regression slope is shown in Fig. 4 for the *F*_*ET*_ and Fig. 5 for the *F*_*NEE*_. These standard errors represent the uncertainty associated with estimation of the isotope ratio of the *F*_*ET*_ and *F*_*NEE*_ within the mixing model framework. The mean of the standard error of the Miller-Tans model slope across all sites ranged from 1.3 to 3.5 (+/− 0.6) for δ^2^H, from 0.2 to 0.5 (+/− 0.1) for δ^18^O, and from 0.2 to 0.7 (+/− 0.1) for δ^13^C.

## Usage Notes

Water isotope ratios of *F*_*P*_ are contained in four CSV files containing time series of δ^2^H (“daily_p_d2H_mean.csv”), δ^2^H (“daily_p_d2H_std.csv”), δ^18^O (“daily_p_d18O_mean.csv”), and δ^18^O (daily_p_d18O_std.csv) ratios corresponding to observed precipitation derived from the 100 ensemble members. A corresponding metadata file (“daily_p_metadata.csv”) describing the quality of each data point is provided as well are the 100 timeseries. The script “Estimate_daily_p_flux_iso.py” will implement the complete statistical downscaling method^22^ with 100 ensemble runs and save daily water isotope time series (mean and standard deviation) which correspond to observed daily precipitation amounts at each NEON site to comma-separated values (CSV) files. The user can change which site data are analyzed and how many ensembles are generated. The user can change the date range they wish to look at. Additionally, the user can change the “precip_filter” variable to change the magnitude of the precipitation events which will be filtered out of the downscaling method and consequently update the output time series and metadata files.

Water isotope ratios of the *F*_*ET*_ and carbon isotope ratios of the *F*_*NEE*_ are contained in three CSV files per time-window (“daily_et_flux_d2H_xxx.csv”, “daily_et_flux_d18O_xxx.csv”, and “daily_nee_flux_d13C_xxx.csv”), where “xxx” is the time-window (“alltime”, “daytime”, and “nighttime”). Each are associated with a corresponding error and metadata file (“daily_et_flux_d2H_xxx_error.csv”, “daily_et_flux_d2H_xxx_metadata.csv”, “daily_et_flux_d18O_xxx_error.csv”, “daily_et_flux_d18O_xxx_metadata.csv”, “daily_nee_flux_d13C_xxx_error.csv”, and “daily_nee_flux_d13C_xxx_metadata.csv”) describing the error estimate of the flux and the quality of each data point. NEON isotope atmospheric isotope ratios were calibrated using the NEONiso package and the *F*_*ET*_ and *F*_*NEE*_ flux isotope composition estimation procedure for δ^13^C, δ^2^H, and δ^18^O were done by leveraging the R scripts “mixing_model_d13C.R”, “mixing_model_d2H.R”, and “mixing_model_d18O.R”, respectively. The script “et_nee_flux.py” takes the output from the mixing-model scripts, reshapes, and assigns quality flags to the data. For the quality flags of *F*_*ET*_ and *F*_*NEE*_ fluxes, the user can adjust the minimum number of the data points used in the Miller-Tans mixing model, adjust the threshold value of the R^2^ regression, and implement other filters besides the IQR filtering.

## Data Availability

Python and R code and generated CSV files are available on HydroShare^35^. For the NEON data processing packages, refer to the NEONiso package^24^ found at on CRAN or at 10.5281/zenodo.3836875.
